# Exceptional Repositioning of Dog Dewormer: Fenbendazole Fever

**DOI:** 10.3390/cimb44100338

**Published:** 2022-10-17

**Authors:** Tania Sultana, Umair Jan, Hyunsu Lee, Hyejin Lee, Jeong Ik Lee

**Affiliations:** 1 Regenerative Medicine Laboratory, Center for Stem Cell Research, Department of Biomedical Science and Technology, Institute of Biomedical Science and Technology, Konkuk University, Seoul 05029, Korea; 2 Department of Veterinary Obstetrics and Theriogenology, College of Veterinary Medicine, Konkuk University, Seoul 05029, Korea

**Keywords:** fenbendazole, microtubule polymerization, self-administration, cancer

## Abstract

Fenbendazole (FZ) is a benzimidazole carbamate drug with broad-spectrum antiparasitic activity in humans and animals. The mechanism of action of FZ is associated with microtubular polymerization inhibition and glucose uptake blockade resulting in reduced glycogen stores and decreased ATP formation in the adult stages of susceptible parasites. A completely cured case of lung cancer became known globally and greatly influenced the cancer community in South Korea. Desperate Korean patients with cancer began self-administering FZ without their physician’s knowledge, which interfered with the outcome of the cancer treatment planned by their oncologists. On the basis of presented evidence, this review provides valuable information from PubMed, Naver, Google Scholar, and Social Network Services (SNS) on the effects of FZ in a broad range of preclinical studies on cancer. In addition, we suggest investigating the self-administration of products, including supplements, herbs, or bioactive compounds, by patients to circumvent waiting for long and costly FZ clinical trials.

## 1. Introduction

Over the past few decades, a considerable amount of research has been conducted on novel oncological therapies; however, cancer remains a major global cause of morbidity and mortality [1]. Developing novel anticancer drugs requires considerable funding for large-scale investigations, experimentation, testing, corroboration, and the subsequent evaluation of efficacy, pharmacokinetics, and toxicity [2]. After this arduous process, only 5% of oncology drugs enter phase I clinical trials [3]. Chemotherapy is considered to be one of the most methodical and vigorous strategies to treat malignant tumors. However, the development of multidrug resistance in patients with cancer receiving traditional chemotherapeutics causes 90% of deaths [4]. Under these circumstances, new therapeutic alternatives are in demand. However, the conventional method of developing new anticancer drugs is onerous, stringent, and costly. The estimated time to discover a single drug candidate is 11.4–13.5 years, and it costs approximately USD 1–2.5 billion to take it through all the obligatory trials required by the U.S. Food and Drug Administration [5].

Drug repurposing or reprofiling has gained recognition and has enabled existing pharmaceutical products to be reconsidered for promising alternative applications, as their pharmacodynamics, pharmacokinetics, and toxicity profile are already well-known in animals and humans [6]. With repurposing, new drugs could be ready for clinical trials faster, reducing development time; this is also economically appealing by expediting integration into medical practice compared with other drug development strategies [7,8]. Examples of high-potential drugs recognized within the Repurposing Drugs in Oncology (ReDO) project include clarithromycin, cimetidine, diclofenac, mebendazole (MBZ), and nitroglycerine [8]. In addition, several antiparasitic drugs that have been in clinical use for decades have been investigated for repurposing in oncology [9]. The repositioning of anthelminthic pleiotropic benzimidazole carbamate (BZ) group drugs such as MBZ, albendazole (ABZ), and flubendazole has recently opened new avenues in cancer treatment owing to their easy access, low cost as generic drugs, and safety in human application [10,11]. Another potent and efficient pharmacological candidate from this group for repurposing as an anticancer drug is fenbendazole (FZ), which is widely used in veterinary medicine to treat parasitic worms including ascarids, whipworms, hookworms, and a single species of tapeworm, *Taenia pisiformis*, in humans and animals [12]. Although there are considerable research and successful cases regarding the anticancer activity and mechanism of action of FZ, there is ongoing social controversy concerning its application in cancer treatment [13,14]. In this review, we summarize the current evidence on the anticancer activity of FZ and the drawbacks of patient self-administration in the treatment regime. 

## 2. Fenbendazole

Methyl N-(6-phenylsulfanyl-1H-benzimidazole-2-yl (FZ) is a safe broad-spectrum antiparasitic drug [12] with proven applications in treating different types of parasitic infections caused by helminths in livestock [15], companion animals [16,17], and laboratory animals [18]. It has a high safety margin with a low degree of toxicity in experimental animals [19] and is well-tolerated by most species, even at sixfold the prescribed dose and threefold the recommended duration. The FZ dosage for dogs is 50 mg/kg/day for 3 days, and it is also safely administered at specific doses in other livestock [20]. Orally administered FZ is poorly absorbed in the bloodstream; thus, it is necessary to retain FZ as long as possible in the rumen, where it is progressively absorbed for higher efficacy. Rarely found side effects are diarrhea and vomiting. Metabolism to its sulfoxide derivative is mainly in the liver, and excretion occurs mainly through feces with a very small amount excreted via urine. 

## 3. Mechanism of Action

**Figure 1:**
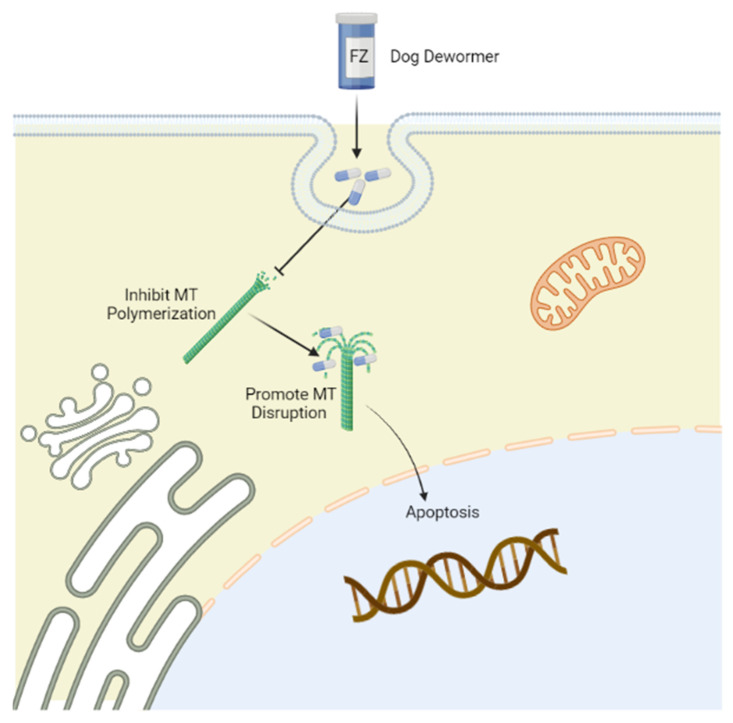
Mechanism of action of fenbendazole (FZ) targeting tubulin. Tubulin is the leading molecular target of FZ, which selectively binds to the β-tubulin of microtubules to disrupt microtubular polymerization, promoting immobilization and the death of parasites. The figure was created using Biorender (https://biorender.com/) (accessed on 15 May 2022).

FZ primarily inhibits tubulin polymerization and promotes microtubular (MT) disruption in parasite cells (Figure 1) [21]. Tubulin, a structural protein of MTs, is the leading molecular target of benzimidazoles [22] and has prominent functions in cell proliferation, motility, division, the intercellular transport of organelles, the maintenance of the cell shape, and the secretion process of cells in all living organisms [23]. By binding with beta-tubulin, FZ blocks MT polymerization in worms and thus perturbs glucose uptake, eventually emptying glycogen reserves and adversely affecting energy management mechanisms. As a result, the whole process eventually contributes to the death of the parasites [20]. Additionally, the poor absorption of FZ from the intestine manifests as reduced levels of drugs and their active ingredients in tissues compared with those within the gut, where the targeted parasites are present [24].

## 4. Anticancer Activity of FZ

MTs are one of the major components of the cytoskeleton, and their role in cell division, the maintenance of cell shape and structure, motility, and intracellular trafficking renders them one of the most important targets for anticancer therapy. Several broadly used anticancer drugs induce their antineoplastic effects by perturbing MT dynamics. A class of anticancer drugs acts by inhibiting MT polymerization (vincristine, vinblastine), while another class stabilizes MTs (paclitaxel, docetaxel), suggesting that FZ could have potential anticancer effects [25,26]. The consequence of disrupting tubulin and dynamic MT stability with these classes of anticancer drugs in dividing cells is apoptosis and metaphase arrest. FZ exhibits moderate MT depolymerizing activity in human cancer cell lines, and has a potent anticancer effect in vitro and in vivo [27]. The antitumor effects of FZ are summarized in Table 1. 

## 5. Anticancer Activity of FZ in Preclinical Models

The anticancer activity of FZ has been investigated in different cell lines. FZ exhibits depolymerizing MT activity toward human cancer cell lines that manifests as a significant anticancer effect in vitro and in vivo. The mechanism of action of the FZ antitumor effect is predominantly the disruption of MT dynamics, p53 activation, and the regulation of genes associated with multiple biological pathways. FZ treatment also causes the depletion of glucose uptake in cancer cells by downregulating key glycolytic enzymes and GLUT transporters [24,27,33]. FZ selectively inhibits the growth of H4IIE cells by upregulating p21, and downregulating cyclins B and D at G1/S and G2/M phases, resulting in apoptosis exclusively in actively growing cells with low confluency, but not in quiescent cells. MAPKs, glucose generation, and ROS are unlikely targets of FZ in H4IIE rat hepatocellular carcinoma cells [32]. Treating human cancer cell lines with FZ induces apoptosis, whereas normal cells remain unaffected. Many apoptosis regulatory proteins such as cyclins, p53, and IκBα that are normally degraded by the ubiquitin–proteasome pathway accumulate in FZ-treated cells. Moreover, FZ produced distinct endoplasmic reticulum (ER) stress-associated genes, such as *ATF3, GADD153*, *GRP78*, *IRE1*α, and *NOXA,* in experimental cells. Thus, FZ treatment in human cancer cells induced decreased mitochondrial membrane potential, ROS production, ER stress, and cytochrome *c* release, eventually leading to cancer cell death [34]. FZ exhibits considerable affinity for mammalian tubulin in MT and is toxic in human cancer cells (H460, A549) at micromolar concentrations. Additionally, FZ exposure causes the mitochondrial translocation of p53, and effectively inhibits the expression of GLUT transporters, glucose uptake, and levels of hexokinase, which is a key glycolytic enzyme potentially linked to p53 activation and the alteration of MT dynamics. Orally administered FZ successfully blocked the growth of human xenografts in a *nu*/*nu* mice model [27]. Moreover, Qiwen et al. reported that FZ treatment is toxic to EMT6 mouse mammary tumor cells in vitro, with toxicity increasing after 24 h incubation with high FZ doses. However, FZ did not alter the dose–response curves for radiation on EMT6 cells under either aerobic or hypoxic conditions [24]. In contrast, Ping et al. reported that FZ or vitamins alone had no growth inhibitory effect on P493-6 human lymphoma cell lines in SCID mice. In combination with vitamin supplements, FZ significantly inhibited tumor growth through its antimicrotubular activity [28]. The effect of a therapeutic diet containing 150 ppm FZ for 6 weeks on the growth of EMT6 mouse mammary tumors in BALB/c mice injected intradermally was examined. The results revealed that the FZ diet did not alter tumor growth, metastasis, or invasion. Therefore, the authors suggested being cautious in applying FZ diets to mouse colonies used in cancer research. [29]. HL-60 cells, a human leukemia cell line, were treated with FZ to investigate the anticancer potential in the absence or presence of N-acetyl cysteine (NAC), an inhibitor of ROS production. NAC could significantly recover the decreased metabolic activity of HL-60 cells induced by 0.5–1 μM FZ treatments. The results proved that FZ manifests anticancer activity in HL-60 cells via ROS production [35]. Ji-Yun also reported the antitumor effect of FZ and paclitaxel via ROS on HL-60 cells at a certain concentration [36]. Moreover, FZ and its synthetic analog induced oxidative stress by accumulating ROS. In addition, FZ activated the p38-MAPK signaling pathway to inhibit the proliferation and increase the apoptosis of HeLa cells. FZ also impaired glucose metabolism, and prevented HeLa cell migration and invasion [37]. Koh et al. found that FZ, MBZ, and oxibendazole possess remarkable anticancer activities at the cellular level via tubulin depolymerization, but their poor pharmacokinetic parameters limit their use as systemic anticancer drugs. However, the same authors assured patients on the application of oxibendazole or FZ as a cancer treatment option [13]. Thus, FZ was investigated using a novel transcriptional drug repositioning approach based on both bioinformatic and cheminformatic components for the identification of new active compounds and modes of action. FZ induced the differentiation of leukemia cells, HL60, to granulocytes, at a low concentration of 0.1 μM within 3 days, causing apoptosis in cancer cells [30]. 

## 6. Complete Cure for Cancer by Self-Administering FZ with Supplements

**Figure 2:**
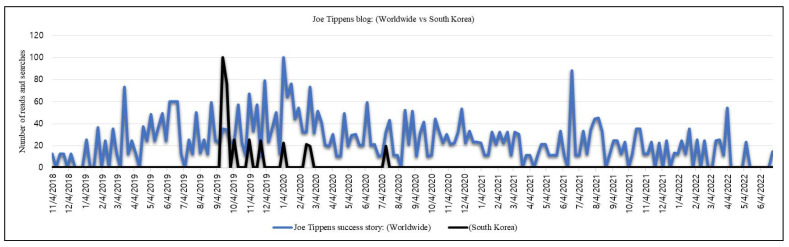
Comparative reading of Joe Tippens’ blog. The number indicates the average number of reads and searches for the use of fenbendazole as an anticancer drug after the release of Tippens’ story. A volume of 100 indicates the highest number, while 0 indicates the lowest. The data were collected using Google Trends and Naver Data Lab (South Korean search engine).

**Figure 3:**
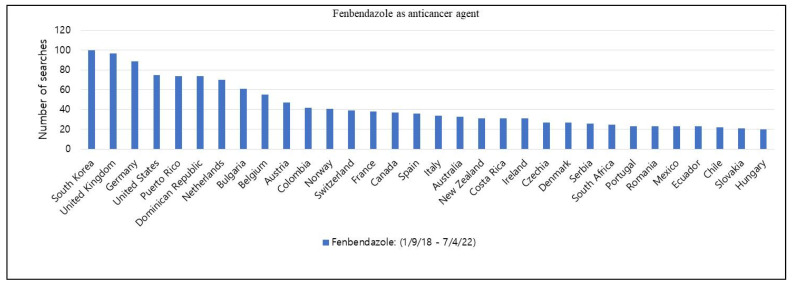
Dissemination of Joe Tippens’ cure story. Thousands of people globally read, listened to, and followed his protocol after his success. The data represent the number of searches conducted by different countries. A value of 100 indicates the highest popularity with the highest number of searches, while 0 indicates the lowest popularity with the lowest number of searches. The data were collected using Google Trends and Naver Data Lab (South Korea search engine).

In August 2016, a businessman from Oklahoma, Joe Tippens, was diagnosed with small-cell lung cancer and underwent a clinical trial under the supervision of his oncologist. He was informed of a short life expectancy from 3 months to 1 year. However, a veterinarian recommended to try FZ with vitamin E supplements, cannabidiol (CBD) oil, and bioavailable curcumin while going through the clinical trial. A positron emission tomography (PET) scan after 3 months did not detect any cancer cells anywhere in his body. Tippens was the only patient cured of cancer among the 1100 patients included in that clinical trial [38]. Tippens shared his success story through Social Network Services (SNS) via a closed group, ‘my cancer story rocks’ (33,900 members) [39], and also in his blog, Get Busy Living (Figure 2), and mentioned at least 60 known FZ success stories [40]. The blog has been read by thousands from 60 different countries, as mentioned in his blog (Figure 3). A protocol is also available on the website recommending FZ 222 mg (1 gm of Panacur™ or Safeguard™) daily for different types of cancer such as colorectal, colon, lung, pancreatic, and prostate cancers, melanoma, lymphoma, and glioblastoma. 

## 7. Liver Injury after Self-Administration of FZ

An 80-year-old patient with advanced nonsmall-cell lung cancer (NSCLC) was treated with pembrolizumab monotherapy. Nine months after treatment initiation, the patient began to present symptoms of severe liver injury. The physical findings and vital signs of the patient were unremarkable. A diligent interview with her and her family disclosed that she had been taking oral FZ for a month solely on the basis of SNS suggesting its effectiveness against cancer. After discontinuing self-administered FZ, the patient’s liver injury gradually improved. Pembrolizumab monotherapy was suspended due to an enlarged tumor in the right upper lobe of her lung [31]. 

## 8. Dissemination of FZ in South Korea

**Figure 4:**
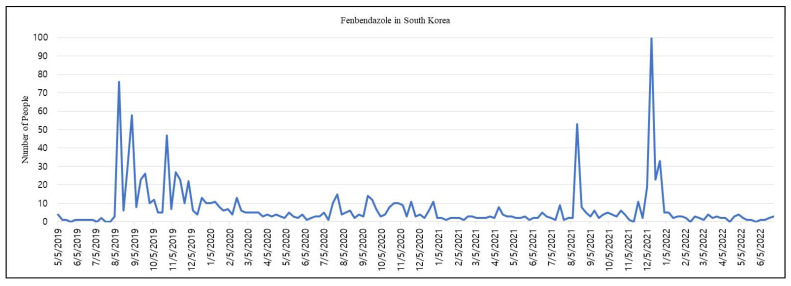
Dissemination of self-administration of fenbendazole. People started following Kim Chul-Min’s story because of celebrity endorsement. The data represent the number of cancer patients or their relatives following Min. A volume of 100 indicates the highest number of searches. The data were collected using Naver Data Lab (a South Korean search engine).

Cancer has been the leading cause of death in Korea since 1983 [41]. The news of Joe Tippens disseminated rapidly among online South Korean cancer patient communities and on social media since September 2019, when a South Korean YouTube channel introduced Joe Tippens’ story on their channel [42]. The video amassed more than 2.4 million views within 3 months. The Korean Medical Association, Korean Pharmaceutical Association, Korean Veterinary Association, and Ministry of Food and Drug Safety have warned patients not to take FZ, as no clinical trials have been conducted on humans. However, disregarding these warnings, dozens of patients with terminal cancer self-administered FZ, regularly uploading videos and reporting on the positive changes observed in their bodies. Vet pharmacies all over South Korea have reported shortages of FZ and frequent inquiries about the availability of this antiparasitic agent by desperate people hoping to cure cancer for themselves or their family members. A South Korean comedian and singer, Kim Chul-Min, who was suffering from Stage 4 lung cancer, joined the FZ bandwagon, reporting that his body pain was alleviated, and his blood test results improved after he had administered FZ in early October 2019 [43] (Figure 4). However, Kim stopped taking FZ after 8 months, mentioning that the drug was ineffective; indeed, FZ use led to serious side effects, recently causing his death [44]. Moreover, an internal medicine specialist in South Korea motivated many cancer patients to urge the government to conduct a clinical trial to determine the oncological efficacy of FZ in humans [45], and also expressed reluctance to use expensive cancer treatments in favor of FZ [46]. The controversy grew further when a Korean oncologist, Kim Ja-young, uploaded a YouTube video favoring FZ titled, ‘Is dog dewormer safe for humans’, depicting the appropriate dosage of the drug; this video reached 60,000–180,000 views and received more than 500 thankful comments within a very short period [47]. However, the medical community has labeled this video as a source of false and exaggerated information.

Another SNS group directly connected to Joe Tippens had pet owners who self-administer FZ using autoprescriptions to treat canine/feline cancer without informing veterinarians [48]. Patients are selling these drugs to each other, and specialized drug sellers are dealing directly with patients, which is strictly prohibited by Korean healthcare laws and regulations. The members are showing eagerness to use FZ or other benzimidazole antiparasitic drugs, including ABZ, MBZ, and FLU, and drugs from different groups such as niclosamide [49], pyrvinium [50], and ivermectin [51] (which is effective against the COVID-19 virus [52]). An increase in the popularity of FZ in South Korea has been observed in online news, YouTube videos, social media, etc. We also discovered that a large number of Google search results regarding the use of FZ against cancer were from South Korea (70.3%; 104,000/148,000).

## 9. Discussion

To accelerate anticancer drug development, agents in clinical use for different indications are screened for repurposing. The approval of such repurposed drugs may be expedited owing to the availability of preclinical and clinical data on pharmacokinetics, toxicities, and regimens [53]. The anticancer activities of several anthelmintics, owing to their microtubule disruption ability, have generated considerable interest. The synergism of BZs, particularly FZ, with many clinically approved anticancer drugs is advantageous in repurposing these drugs. In the veterinary field, pets are often prescribed human medicines, since those available for companion animals are insufficient to cover all animal diseases. Likewise, commercially available animal drugs might be utilized in human medicine as long as human clinical trials have been conducted. However, globally, patients with cancer use SNS to repurpose veterinary medicines as anticancer drugs, following dosage regimens provided by self-cured patients. Sources of medical information on SNS are often unproven, and it is challenging for nonmedical professionals to precisely select and filter complex medical information. Considerable effort is required to set the effective dosages of FZ for humans. Moreover, if a patient self-administers a veterinary dewormer with an established anticancer drug while participating in a clinical trial, the outcome of the clinical trial could be altered completely, resulting in huge economic and time losses. Therefore, the reason for the therapeutic or adverse effects of the trialed drug in the clinical trial remains equivocal, and oncologists are confounded by their lack of knowledge regarding the self-administration of the dewormer (FZ) by patients. 

FZ began to gain popularity as a human anticancer drug in South Korea in September 2019. The serious consequences of such events will likely emerge soon in South Korea. As the number of success stories being published online increases, the uninformed self-administration of FZ will also continue to increase in South Korea. Any prohibition by the government or medical doctors will likely not be followed by desperate patients with cancer. The resulting situation would lead to possible global FZ self-administration by patients with cancer. Therefore, an alternative must be offered to relieve patients from the long and expensive wait for FZ clinical trials in humans. 

Indeed, patients with cancer and their families desperately seek remedies to treat the disease, but administering drugs with limited or no human safety profiles is a concern for clinicians. However, there are many exceptions for investigational drugs in clinical trials. There is a plethora of evidence that several BZ carbamates, particularly FZ, show anticancer potential in vivo, in vitro, and in silico. The role of MT is well-characterized, and the mechanism of action of MT disruptors such as FZ is similar to that of major chemotherapeutic agents such as vinblastine and vincristine. Nevertheless, nonmedical professionals cannot make appropriate medical judgments regarding the best usage of FZ. Therefore, we argue in favor of FZ owing to its well-established pharmacokinetics, excellent toxicity profile, and low cost, and suggest that an initial assessment of patients by interdisciplinary researchers such as veterinarians, oncologists, and pharmacologists be conducted to determine the best initial dosage and facilitate clinical trials on FZ in the near future.

## 10. Conclusions

Comprehensive verification through evidence-based medicine is crucial in reducing needless confusion in healthcare. Despite the potential anticancer capabilities of FZ, its pharmacokinetics, safety, and tolerance profiles in humans must be confirmed in extensive clinical trials before it can be used in any therapeutic context. Experts must further attempt to provide patients with reliable medical information. 

## 11. Materials and Methods

### Process of Article Selection

We used search engines PubMed, Naver, and Google Scholar to obtain publications and articles investigating the anticancer activity of FZ in cell lines, animal tumor models, and clinical trials. For a report to be included in this review, it had to have contained FZ or BZ carbamate in either the heading or abstract. Keywords ‘BZ carbamate’ and ‘FZ’ combined with ‘MT polymerization,’ ‘cancer,’ and ‘antiparasitic’ were used to generate the list. Review articles were not included in our survey. All the relevant articles were initially recognized from the heading and abstract, followed by an additional examination to confirm whether the research conducted using FZ drugs pertained to cell lines or human or animal subjects. We ensured that the conducted search technique was not encyclopedic, as many publications are not included in PubMed, Naver, or Google Scholar. We evaluated the selected studies by assessing different characteristics such as cell lines and source, animal model, cancer type, and target pathway.

## Figures and Tables

**Table 1:** Anticancer activity of fenbendazole.

Cell Source	Cell Lines	Species	Procedure of Study	Cancer Type	Target Pathway	Reference
Human	A549, H460, and H1299	-	In vitro	Lung cancer	Tubulin polymerization	[13]
Human	H460 and A549	Mice	In vivo and in vitro	NSCLC	Endoplasmic reticulum stress, ROS production, decreased mitochondrial membrane potential, and cytochrome c release	[27]
Human	P493-6 B	SCID mice	In vivo and in vitro	Lymphoma	Tubulin disruption	[28]
Mice	EMT6	BALB/c Rw mice	In vivo	Lung cancer	Tubulin disruption	[29]
Human	BMSC, HFF, and HL60	Mice	In vivo and in vitro	Leukemia	Granulocyte differentiation and PI3K/AKT, JAK/STAT, and MAPK pathways	[30]
-	-	Human	In vivo	NSCLC	-	[31]
Rat	H4IIE	Mice	In vitro	Hepatocellular Carcinoma	MAPKs, glucose generation, and reactive oxygen species (ROS)	[32]

BMSC, bone marrow-derived mesenchymal stem cells; HFF, human foreskin fibroblast cells; NSCLC, nonsmall-cell lung cancer.

## Data Availability

Not applicable.
